# Supplementary material for: Time Trends in Treatment Strategies and Survival of Older versus Younger Patients with Synchronous Metastasised Melanoma—A Population-Based Study in the Netherlands Cancer Registry
Source: Cancers (Basel). 2022 Oct 7;14(19):4904. doi: 10.3390/cancers14194904 (PMC9563569; doi:10.3390/cancers14194904)
Supplement: Supplementary file 1 [file cancers-14-04904-s001.zip › cancers-1906690-supplementary.pdf]

Table S1. numbers of patients treated with immunotherapy.

Year	<65		65-75		>75	
	n	*(%)*	*n*	*(%)*	*n*	*(%)*
2000	6	(11.8)	0	(0.0)	0	(0.0)
2001	4	(7.0)	0	(0.0)	0	(0.0)
2002	3	(4.3)	0	(0.0)	0	(0.0)
2003	1	(1.5)	1	(4.8)	0	(0.0)
2004	1	(1.6)	0	(0.0)	0	(0.0)
2005	1	(1.3)	0	(0.0)	0	(0.0)
2006	1	(1.6)	0	(0.0)	0	(0.0)
2007	2	(2.6)	0	(0.0)	0	(0.0)
2008	1	(1.3)	0	(0.0)	0	(0.0)
2009	1	(1.2)	0	(0.0)	0	(0.0)
2010	4	(4.1)	1	(3.1)	1	(3.2)
2011	9	(10.2)	0	(0.0)	0	(0.0)
2012	5	(7.1)	1	(2.3)	1	(2.9)
2013	5	(6.8)	3	(7.0)	0	(0.0)
2014	22	(25.3)	12	(25.0)	2	(5.6)
2015	37	(39.4)	21	(43.8)	8	(23.5)
2016	30	(34.1)	17	(27.9)	14	(31.8)
2017	32	(42.7)	23	(37.7)	7	(13.5)
2018	44	(50.0)	31	(50.8)	18	(35.3)

Table S2. numbers of patients treated with targeted therapy.

Year	<65		65-75		>75	
	n	*(%)*	*n*	*(%)*	*n*	*(%)*
2000	0	(0.0)	0	(0.0)	0	(0.0)
2001	0	(0.0)	0	(0.0)	0	(0.0)
2002	0	(0.0)	0	(0.0)	0	(0.0)
2003	0	(0.0)	0	(0.0)	0	(0.0)
2004	0	(0.0)	0	(0.0)	0	(0.0)
2005	0	(0.0)	0	(0.0)	0	(0.0)
2006	1	(1.6)	0	(0.0)	0	(0.0)
2007	0	(0.0)	0	(0.0)	0	(0.0)
2008	1	(1.3)	0	(0.0)	0	(0.0)
2009	0	(0.0)	0	(0.0)	0	(0.0)
2010	1	(1.0)	0	(0.0)	0	(0.0)
2011	7	(8.0)	1	(2.6)	0	(0.0)
2012	9	(12.9)	8	(18.2)	5	(14.7)
2013	18	(24.3)	9	(20.9)	2	(5.9)
2014	25	(28.7)	9	(18.8)	4	(11.1)
2015	25	(26.6)	13	(27.1)	4	(11.8)
2016	27	(30.7)	14	(23.0)	3	(6.8)
2017	27	(36.0)	9	(14.8)	5	(9.6)
2018	25	(28.4)	13	(21.3)	5	(9.8)
